# Correlates of Suicide Attempts in Filipino Youths: An Analysis Based on the 2015 Global School-Based Student Health Survey

**DOI:** 10.7759/cureus.18100

**Published:** 2021-09-19

**Authors:** Jasmin Gange Lagman, Michael Gara, Raman Baweja, Wun Jung Kim

**Affiliations:** 1 Department of Psychiatry and Behavioral Health, Penn State Health Milton S. Hershey Medical Center, Hershey, USA; 2 Department of Psychiatry, Rutgers Robert Wood Johnson Medical School, Piscataway, USA

**Keywords:** philippines, suicide attempt, methamphetamine, global school-based health survey, adolescent

## Abstract

There is a lower incidence of suicide in the Philippines compared to other developed/developing countries, but the trend has been increasing. This study aims to identify the correlates of suicide attempts in Filipino youth using the World Health Organization's 2015 Global School-Based Health Survey. All schools in the Philippines with grades 7-10 were included. A stratified sampling design was used, and participants were randomly sampled. Of the 8,761 students who participated in the survey, 16.2% have attempted suicide at least once in the past 12 months. The factors that increased the risk of suicide attempts include female gender, being physically attacked, getting bullied, feeling lonely, poor sleep, having few close friends, smoking, alcohol use, less physical activity, use of amphetamine/methamphetamine, and less parental supervision. The use of methamphetamine/amphetamine is the single best risk factor of suicide attempts among Filipino youth with OR= 4.6; 95% CI [3.8, 5.6].

## Introduction

Suicide is a global phenomenon and is the fourth leading cause of death among 15-29 years old worldwide [1, 2]. In the United States (US), suicide is the second leading cause of death among youths ages 15-19 years old [3]. In contrast to other developed/developing countries, there is a lower incidence of suicide in the Philippines with a crude suicide rate of 2.2 per 100,000 population compared to 9/100,000 population globally [2]. As a predominantly Catholic country, there may be under-reporting or misclassification of suicide as injury of undetermined intent [4]. Over recent years, the incidence of suicide in the Philippines has been increasing and there is an urgent call to understand suicide behavior and the need to establish prevention strategies [4]. It is imperative to study the trends of suicide and understand risk factors among adolescents in the Philippines.

There are various risk factors including psychosocial and biological factors that have been identified. Those who have attempted suicide are at high risk for a later suicide completion especially during the following year of their first attempt [5]. Treatment guideline has highlighted various risk factors including depression, family history of suicide attempts, exposure to violence, impulsivity, aggressive or disruptive behavior, access to firearms, bullying, feelings of hopelessness or helplessness, and acute loss or rejection [6]. The American Academy of Pediatrics (AAP) Committee on Adolescence listed additional risk factors for suicide and suicide attempts which include history of adoption, male gender, parental mental health problems, lesbian, gay, bisexual, or questioning sexual orientation, transgender identification, a history of physical or sexual abuse, pathologic internet use, and non-suicidal self-injurious behaviors [7]. Agitation, intoxication, and recent stressful life events were considered as immediate risk factors while bullying, impaired parent-child relationship, living outside of the home, difficulties in school, and neither working nor attending school were social and environmental risk factors [7]. However, information about the specific risk factors in a defined geographical area such as the Philippines is limited. Given the increased suicide rates in this country, there is an urgent need to understand the trends and to identify risk factors for youth in this specific region. The goal of this study is to identify the correlates of suicidal ideation and suicide attempts in Filipino youths using the 2015 Global School-Based Health Survey (GSHS).

This article was previously presented as a poster at the 2018 American Academy of Child and Adolescent Psychiatry Meeting at Seattle, USA on October 27, 2018.

## Materials and methods

Sample

The World Health Organization (WHO), in collaboration with Joint United Nations Programme on Human Immunodeficiency Virus/Acquired Immune Deficiency Syndrome, United Nations Educational, Scientific and Cultural Organization and the United Nations International Children's Emergency Fund, with assistance from US Centers for Disease Control and Prevention started the GSHS and periodically monitors the prevalence of important risk behaviors and protective factors among students [8]. It includes possible risk factors affecting suicidal ideation and suicide attempts among Filipino students. The Philippines is one of the 94 countries that participates in this survey [8]. This study is a secondary analysis of the most recent public data from the Philippine GSHS. Ethics review is not required since data is publicly available. The survey was conducted on students who were 13-17 years of age. All schools in the Philippines with grades 7-10 were included in the sampling range.

Instrument

The survey was conducted in March 2015. The students completed a self-administered questionnaire that contained 80 multiple-choice questions from three components: core modules, core expanded questions, and country-specific questions. It measures the behaviors and protective factors related to the leading causes of morbidity and mortality among children and adults. The questions were translated into the Filipino language [9]. To determine suicidal attempts, this question was considered: “During the past 12 months, how many times did you actually attempt suicide?” (See Appendix A [10]). Risk factors and protective factors for suicide attempts were assessed with 22 questions, including demographics, psychosocial stressors, and substance abuse (See Appendix B [10]).

Design and statistical analyses

A stratified sampling design was used, in which 96 schools serving 7th through 10th graders were randomly sampled (N = 8761). Then, classes were randomly sampled from the 90 schools which participated in the survey [9]. Weighting was used to allow results to be generalized to the entire population of students, not just those who took the survey. Weighting allows one student to represent many other students with similar demographic characteristics and is necessary for all sample-based surveys since data are not collected from all members of the target population. Weighting accounts for the probability of selection of schools and classrooms, non-responding schools and students, and distribution of the population by grade and sex. To incorporate subject weights, strata (school), and cluster (classroom) in cross-tabulation and logistic regression analyses, PROC SURVEYFREQ and PROC SURVEYLOGISTIC in SAS Version 9.4 (SAS Institute Inc., Cary, NC) were used to analyze the data. Logistic regression was done in two stages, the first to eliminate predictor variables associated with p-levels >= 0.005. Variance estimation entailed Taylor Series Linearization (TSL) with a finite population correction term.

## Results

Demographic

There was a total of 8,761 students who participated in this survey from 90 schools and were equally represented by male (49.5%) and female (50.5%). Most of the respondents came from public schools, and the highest respondents were from grade 7 and grade 8. Table 1 shows that 16.2% had at least one suicide attempt in the past 12 months.

**Table 1:** Frequency of suicide attempts.

Number of suicide attempts	N (%)
1	814 (9.97%)
2-3	345 (4.47%)
4-5	88 (1.02%)
6 or >6	60 (0.74%)

Risk factors of suicide attempts

Among the 16 variables identified as risk or protective factors of suicide attempt, 11 variables were significant. The final logistic regression of the significant variables was presented in Table 2. There were 11 predictors for suicide attempts that were significant in this study. The top four includes the use of amphetamine or methamphetamine (OR = 4.6; 95% CI [3.8, 5.6]), having fewer friends (OR = 2.7; 95% CI [2.0, 3.6]), smoking (OR = 2.4; 95% CI [1.3, 1.9]) and feeling lonely (OR = 2.1; 95% CI [1.7, 2.5]). Other significant predictors for suicide attempt among Filipino youths include getting bullied (OR = 1.9; 95% CI [1.7, 2.2]), alcohol drinking in the past 30 days (OR = 1.6; 95% CI [1.3, 1.9]), females (OR = 1.6; 95% CI [1.4, 1.9]), being physically attacked (OR = 1.6; 95% CI [1.5, 1.8]), getting worried and cannot sleep (OR = 1.5; 95% CI [1.2, 2.0]), having less physical activity (OR = 1.2; 95% CI [1.1, 1.3]), and less parental supervision (OR = 1.2; 95% CI [1.0, 1.3]).

**Table 2:** Risk factors of suicide attempt.

Effect	Chi-Square	p-value	Odds Ratio (95% CI)
Age	11.8908	0.1042	NS
Female gender	19.8006	<0.0001	1.6 (1.4-1.9)
Lack of food at home	2.5348	0.6384	NS
Physically attacked	39.9025	<0.0001	1.6 (1.5-1.8)
Bullied	57.7184	<0.0001	1.9 (1.7-2.2)
Lonely	45.8517	<0.0001	2.1 (1.7-2.5)
Worried and can’t sleep	29.2990	<0.0001	1.5 (1.2-2.0)
Few friends	16.4709	0.0009	2.7 (2.0-3.6)
Has tried smoking	51.8254	<0.0001	2.4 (1.9-2.9)
Smoking in past 30 days	6.5438	0.3651	NS
Has tried drinking alcohol	10.9625	0.1403	NS
Drinking in past 30 days	354.5912	<0.0001	1.6 (1.3-1.9)
Got in trouble due to drinking	5.7197	0.01261	NS
Has tried using illegal drugs	8.9367	0.2572	NS
Has used marijuana in past 30 days	8.1722	0.0855	NS
Used Amphetamine or Methamphetamine	16.6219	0.0023	4.6 (3.8-5.6)
Less physical activity	24.7453	0.0008	1.2 (1.1-1.3)
Attended physical education class	1.2337	0.9416	NS
Truancy	4.8407	0.3040	NS
Helpful school peers	11.9009	0.0181	NS
Less parental supervision	30.7770	<0.0001	1.2 (1.0-1.3)
Parents understanding students’ concerns	11.4590	0.0219	NS
Parental awareness of students’ free time	0.8587	0.9304	NS

## Discussion

This study examined possible factors associated with Filipino youth’s suicidal attempts from the most recent public data of the Philippine Global School-Based Health Survey. In the current study, the incidence of suicide attempts was 16.2% in the Philippines, which is significantly higher than the 9.7% mean proportion of adolescents reporting history of suicide attempts in 2005 based on international population studies [11]. Suicidal ideation among Filipino youths ages 13-15 years has declining trends from 16.8% in 2007, 16.3% in 2011 to 11.5% in 2015, while suicide attempts had opposite trends with a significant increase from 12.9% to 17% [12]. The recent increase in suicide attempts in the Philippines is concerning.

Similar to previous reports, youths from the Philippines reported similar risk factors including female gender, being physically attacked, getting bullied, feeling lonely, getting worried and can’t sleep, having less physical activity, smoking, alcohol drinking, use of amphetamines or methamphetamine, and less parental supervision [13-20], consistent with other studies from developing and developed countries about higher suicidality in females than males [14, 15]. However, males have a higher completed suicide than females due in part to the use of more lethal methods in their attempts [4, 15]. Bullying is also a well-documented risk factor for suicidal ideation and behavior [16, 17]. Holt et al. described that any involvement in bullying in any capacity is associated with suicidality and being a victim is associated with the greatest risk [16]. Similarly, depressive disorders and symptoms like feelings of loneliness, sleeping problems, decreased energy and less physical activity are also associated with worsening suicidal behaviors [6, 7]. Substance use is another strong predictor of suicidal behaviors [13, 15]. In Scherrer’s [18] study, smoking was associated with suicide ideation, plan and attempt for nicotine-dependent smokers.

The most recent data from the International Narcotics Control and Strategy Report shows that methamphetamines and cannabis continue to be the two preferred illegal substances in the Philippines [21]. It is worth mentioning that even though only 4.9% of Filipino students used methamphetamines in this study, it showed that it is the strongest risk factor of suicide attempt. The increased risk by 4-5 times with the use of amphetamine/methamphetamine compared with other risk factors was alarming. Several studies have found a similar association between methamphetamine use and suicidal behavior. In the study by Marshall et al. [19], there was an 80% risk of suicide attempts among injected methamphetamines users as compared to non-users. This increased risk might be related to the combination of various mechanisms [19]. For example, adolescents who use methamphetamines were likely to experience depression, suicide ideation, school and legal problems [22]. Also, chronic methamphetamine use can lead to dopamine transporter reduction and lower dopamine level, which is associated with suicidal behaviors [23, 24]. In the Philippines crystal methamphetamine known by its street name “shabu”, was the most commonly used illegal drug and the most significant drug problem in the country at the time this survey was completed [25]. Furthermore, some young Filipino men view crystal methamphetamine as a performance enhancer [26]. Marijuana was the second widely used illegal drug but the most frequently used by adolescents in the Philippines at the time of the survey [21]. In this study, the use of marijuana was not associated with suicide attempt, which was in contrast to other studies from different countries [27, 28].

This study has several limitations. The result cannot represent all youths ages 13-17 years as participants in this study are restricted to school enrollees. Not attending school is a risk factor for suicidal behavior [6]. Since the authors evaluated possible risks factors for suicide attempt based on the available data published publicly, information about many other important variables like psychiatric diagnoses, academic stress, and trauma history are not available. Another limitation is the cross-sectional design of this study which does not give the causal relationships between the variables. This study is based on a self-administered questionnaire completed by youths and recall bias is another limitation of this study. Future studies should focus on exploring different risk factors in different grade levels and also include other psychosocial factors such as access to guns. Comparative studies between developing and developed countries will be helpful to identify different risk factors for youth suicide.

## Conclusions

The findings in this study support the data from previous studies on risk factors affecting suicide attempts among adolescents. They include many consistent findings, such as female gender, social isolation/loneliness, substance abuse, trauma, and lack of parental involvement. Of importance is the finding that the use of amphetamine/methamphetamine was associated with higher suicidal behaviors among Filipino youths. This is concerning given methamphetamine is the most common illegal drug abused in the country. As methamphetamine is widely abused in other Asian countries and its abuse is rising in other parts of the world, this finding has a significant implication for public health policy. The study findings can help the policymakers, public health and school officials adopt and implement policies geared towards child and adolescent mental health to help address the factors correlated with suicidal behaviors among in-school youths in the Philippines and other countries. There is also a need for increased awareness and education to parents/families and teachers about mental health and substance use among youths.

## Appendices

APPENDIX A

This is the question taken from the Global School-based Student Health Survey (GSHS) 2015 Philippines GSHS Questionnaire to ask for history of suicide attempt. Zero times was equivalent to “No” in the statistical analysis while one time to six or more was equivalent to “Yes”.

During the past 12 months, how many times did you actually attempt suicide?

A. 0 times

B. 1 time

C. 2 or 3 times

D. 4 or 5 times

E. 6 or more times

Source: Philippines 2015 GSHS Questionnaire [10].

APPENDIX B

The following are the questions taken from the Global School-based Student Health Survey (GSHS) 2015 Philippines GSHS Questionnaire to assess for significant risk factors for suicide attempt among Filipino Youths.

1. How old are you?

A. 11 years old or younger

B. 12 years old

C. 13 years old

D. 14 years old

E. 15 years old

F. 16 years old

G. 17 years old

H. 18 years old or older

2. What is your sex?

A. Male

B. Female

3. During the past 30 days, how often did you go hungry because there was not enough food in your home?

A. Never

B. Rarely

C. Sometimes

D. Most of the time

E. Always

4. During the past 12 months, how many times were you physically attacked?

A. 0 times

B. 1 time

C. 2 or 3 times

D. 4 or 5 times

E. 6 or 7 times

F. 8 or 9 times

G. 10 or 11 times

H. 12 or more times

5. During the past 30 days, on how many days were you bullied?

A. 0 days

B. 1 to 2 days

C. 3 to 5 days

D. 6 to 9 days

E. 10 to 19 days

F. 20 to 29 days

G. All 30 days

6. During the past 12 months, how often have you felt lonely?

A. Never

B. Rarely

C. Sometimes

D. Most of the time

E. Always

7. During the past 12 months, how often have you been so worried about something that you could not sleep at night?

A. Never

B. Rarely

C. Sometimes

D. Most of the time

E. Always

8. How many close friends do you have?

A. 0

B. 1

C. 2

D. 3 or more

9. How old were you when you first tried a cigarette?

A. I have never smoked cigarettes

B. 7 years old or younger

C. 8 or 9 years old

D. 10 or 11 years old

E. 12 or 13 years old

F. 14 or 15 years old

G. 16 or 17 years old

H. 18 years old or older

10. During the past 30 days, on how many days did you smoke cigarettes?

A. 0 days

B. 1 or 2 days

C. 3 to 5 days

D. 6 to 9 days

E. 10 to 19 days

F. 20 to 29 days

G. All 30 days

11. How old were you when you had your first drink of alcohol other than a few sips?

A. I have never had a drink of alcohol other than a few sips

B. 7 years or younger

C. 8 or 9 years old

D. 10 or 11 years old

E. 12 or 13 years old

F. 14 or 15 years old

G. 16 or 17 years old

H. 18 years old or older

12. During the past 30 days, on how many days did you have at least one drink containing alcohol?

A. 0 days

B. 1 or 2 days

C. 3 to 5 days

D. 6 to 9 days

E. 10 to 19 days

F. 20 to 29 days

G. All 30 days

13. During your life, how many times have you got into trouble with your family or friends, missed school, or got into fights, as a result of drinking alcohol?

A. 0 times

B. 1 or 2 times

C. 3 to 9 times

D. 10 or more times

14. How old were you when you first used drugs?

A. I have never used drugs

B. 7 years old or younger

C. 8 or 9 years old

D. 10 or 11 years old

E. 12 or 13 years old

F. 14 or 15 years old

G. 16 or 17 years old

H. 18 years old or older

15. During the past 30 days, how many times have you used marijuana?

A. 0 times

B. 1 or 2 times

C. 3 to 9 times

D. 10 to 19 times

E. 20 or more times

16. During your life, how many times have you used amphetamines or methamphetamines, also called shabu?

A. 0 times

B. 1 or 2 times

C. 3 to 9 times

D. 10 to 19 times

E. 20 or more times

17. During the past 7 days, on how many days were you physically active for a total of at least 60 minutes per day? Add up all the time you spend in any kind of physical activity each day.

A. 0 days

B. 1 day

C. 2 days

D. 3 days

E. 4 days

F. 5 days

18. During this school year, on how many days did you go to physical education (PE) class each week?

A. 0 days

B. 1 day

C. 2 days

D. 3 days

E. 4 days

F. 5 or more days

G. 6 days

H. 7 days

19. During the past 30 days, on how many days did you miss classes or school without permission?

A. 0 days

B. 1 to 2 days

C. 3 to 5 days

D. 6 to 9 days

E. 10 or more days

20. During the past 30 days, how often were most of the students in your school kind and helpful?

A. Never

B. Rarely

C. Sometimes

D. Most of the time

E. Always

21. During the past 30 days, how often did your parents or guardians understand your problems and worries?

A. Never

B. Rarely

C. Sometimes

D. Most of the time

E. Always

22. During the past 30 days, how often did your parents or guardians really know what you were doing with your free time?

A. Never

B. Rarely

C. Sometimes

D. Most of the time

E. Always

Source: Philippines 2015 GSHS Questionnaire [10].
